# Impact of Early Initiation of Renal Replacement Therapy on Renal Recovery and Mortality in Critically Ill Patients with Acute Kidney Injury: A Prospective Cohort Study

**DOI:** 10.3390/biomedicines13112575

**Published:** 2025-10-22

**Authors:** Rayane Alves Moreira, Sheila Borges, Sarah Lopes da Silva Borges, Marcia Cristina da Silva Magro

**Affiliations:** 1 Graduate Program in Nursing, University of Brasília (UnB), Brasilia 70910-900, Brazil; 2 Health Sciences and Technologies Graduate Program, University of Brasília (UnB), Brasilia 72220-275, Brazil; 3 Centro Universitário Projeção (Uniprojeção), Brasilia 72220-275, Brazil

**Keywords:** acute kidney injury, renal replacement therapy, intensive care units, renal function recovery, dialysis timing, mortality

## Abstract

**Background/Objectives**: Optimal timing to initiate renal replacement therapy (RRT) in critically ill patients with acute kidney injury (AKI) remains uncertain. This study evaluated the impact of early RRT initiation (<24 h after severe AKI diagnosis) on renal recovery and mortality in intensive care unit (ICU) patients. **Methods:** This prospective cohort included 119 patients with dialysis-requiring AKI admitted to two ICUs between December 2022 and December 2024. Patients were categorized according to RRT initiation timing (early < 24 h or delayed >24 h). Mortality (at 20, 30, and 76 days) and renal recovery (at 3, 10, and 30 days) were assessed using Kaplan–Meier curves and a log-rank test. Moreover, multivariate logistic regression was performed to identify factors associated with renal recovery. **Results**: Early RRT was initiated in 39 (32.8%) patients, and delayed RRT was initiated in 80 (67.2%). The early group had higher clinical severity (SOFA: 10 vs. 7; *p* = 0.016). Mortality did not differ between the groups (log-rank *p* = 0.396, 0.742, and 0.932 at 20, 30, and 76 days, respectively). However, early renal recovery (within 3 days) was more frequent in the early group (51.3% vs. 27.5%; *p* = 0.010), and early RRT was an independent predictor of this outcome (OR 3.26; 95% CI: 1.37–7.75 *p* = 0.008). **Conclusions**: Early RRT did not reduce mortality but was associated with improved early renal recovery in critically ill patients with AKI.

## 1. Introduction

The incidence of dialysis-requiring acute kidney injury (AKI) has been increasing globally and is associated with high mortality, particularly due to the severity of illness and advanced age of patients admitted to intensive care units (ICUs) [1].

Among critically ill patients in ICUs, approximately 50% develop AKI, and 4–15% progress to severe AKI requiring renal replacement therapy (RRT). Hospital mortality among patients with dialysis-requiring AKI (AKI-D) can reach 50–80%. [2]. In this subgroup, 13–29% remain dialysis-dependent at discharge, and 10–30% of those are subsequently diagnosed with end-stage kidney disease (ESKD) [3].

A systematic review and meta-analysis highlighted that among patients with severe AKI, RRT does not appear to impact mortality but may delay renal recovery [4]. In this context, evidence from additional meta-analyses shows controversy regarding the association between the type of renal support and the likelihood of renal function recovery [5].

In severe AKI, both the timing and the long-term indication for RRT may influence renal recovery [6]. Clinically, the presence of hypervolemia, hyperkalemia, metabolic acidosis, or uremia often indicates the need for early initiation of RRT [7]. Conversely, in the absence of these complications, determining the optimal timing for RRT initiation remains a clinical challenge [8,9].

The optimal timing for initiating RRT in critically ill patients with AKI remains a matter of debate. Randomized clinical trials have shown conflicting results, while the ELAIN trial [10] demonstrated reduced mortality with early RRT initiation (< 24 h). Other large multicenter trials, such as IDEAL-ICU [8], AKIKI [11], AKIKI II [12], and STAART-AKI [13], found no significant difference in mortality between early and delayed strategies. Additionally, systematic reviews and meta-analyses have highlighted this heterogeneity, suggesting that early RRT initiation does not have a consistent impact on mortality, renal recovery, or dialysis dependence [14,15].

The Acute Disease Quality Initiative (ADQI), which convened international experts to develop practical recommendations on the use of renal replacement therapy (RRT) in critically ill patients with AKI, based its conclusions on a thorough literature review and structured deliberation. The consensus suggests that early initiation of acute renal support may be associated with improved renal recovery and increased patient survival. However, the decision to initiate RRT should consider key factors, such as the availability of appropriate equipment, supplies, and trained personnel to ensure the safe and effective delivery of RRT. In resource-limited settings, such constraints may significantly influence AKI management [16].

The optimal timing for initiating RRT should take into account the patient’s clinical condition, aiming to reduce mortality, enhance the likelihood of renal recovery, and decrease the risk of progression to chronic kidney disease (CKD) [9,17,18].

One of the main goals established by the International Society of Nephrology for 2025 is to eliminate, or at least reduce, preventable deaths caused by AKI worldwide. To achieve this objective, several strategic pillars have been proposed, including the appropriate prescription of RRT tailored to each patient’s individual needs. This includes considerations such as the optimal timing for initiation, modality, dosage, patient-specific characteristics, and availability of healthcare resources [19]. Therefore, this study aimed to evaluate whether the early initiation of renal replacement therapy influences outcomes in critically ill patients with AKI.

## 2. Materials and Methods

### 2.1. Study Design and Settings

This prospective cohort study, conducted in accordance with the STROBE guidelines, was carried out between December 2022 and December 2024 in two intensive care units, one from a private hospital and the other from a public teaching hospital in Brasília, Federal District, Brazil. In both institutions, intermittent and continuous renal replacement therapies were available as treatment options for patients with severe acute kidney injury. The study period was defined to ensure the inclusion of a representative sample with clinical relevance for dialysis-requiring AKI management.

Demographic, clinical, and outcome variables were collected from the patients’ individual electronic medical records, which were accessible only to authorized healthcare professionals.

This study was approved by the Research Ethics Committee at the Faculty of Health Sciences at the University of Brasília (UnB) and authorized by the administration of the participating private hospital. Ethics approval code: CAEE 61211422.1.0000.0030; protocol number: 5.700.052. The study was conducted in full compliance with Resolution No. 466, dated 12 December 2012, of the Brazilian National Health Council (CNS), ensuring the confidentiality and protection of participants’ data. All participants were informed about the purpose of the study and provided written informed consent prior to enrollment.

### 2.2. Study Population and Inclusion and Exclusion Criteria

**Figure 1:**
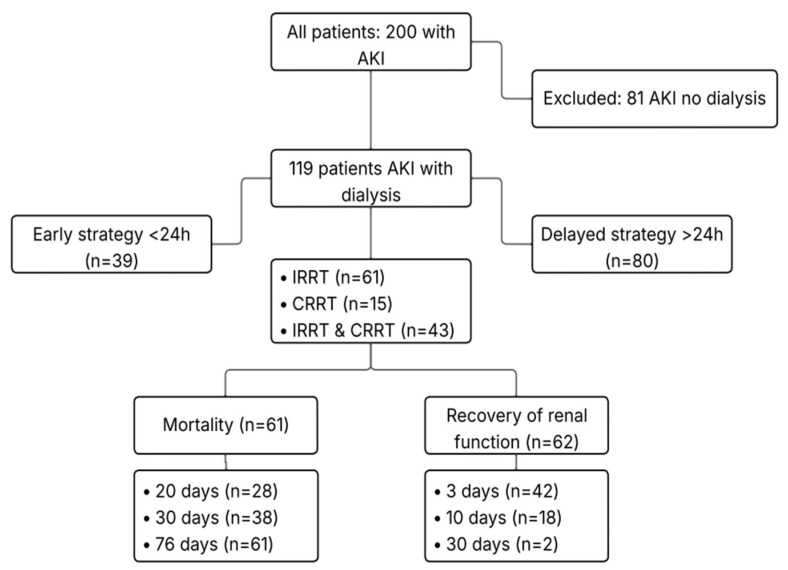
STROBE flowchart for critically ill patients with acute kidney injury (AKI). A total of 200 adult patients with AKI, admitted to two intensive care units between December 2022 and December 2024, were evaluated. Among them, 81 patients with AKI who did not require dialysis were excluded. The remaining 119 patients with dialysis-dependent AKI were analyzed and stratified according to the timing of initiation of renal replacement therapy (RRT): early strategy (< 24 h, n = 39) and delayed strategy (>24 h, n = 80). Patients underwent intermittent RRT (IRRT, n = 61), continuous RRT (CRRT, n = 15), or both modalities (IRRT & CRRT, n = 43). Renal function recovery occurred in 62 patients: at 3 days (n = 42), 10 days (n = 18), and 30 days (n = 2). Overall mortality was observed in 61 patients: at 20 days (n = 28), 30 days (n = 38), and 76 days (n = 61).

All patients or their legal representatives signed the informed consent form. A total of 200 patients with AKI were followed, of whom 119 required dialysis support (STROBE flowchart; Figure 1).

The sample size calculation was based on a power of 80% and an estimated mortality rate of 20%, as reported by Hoste et al. [2]. Accordingly, a minimum of 128 participants was calculated. However, accounting for an anticipated 20% dropout rate, at least 154 participants were considered necessary according to the a priori calculation.

Eligibility criteria included patients aged ≥ 18 years with dialysis-requiring AKI, defined and classified according to the KDIGO (2012) criteria, who received intermittent renal replacement therapy (up to 6 h) and/or continuous therapy during their ICU stay.

Patients were excluded if they had an estimated baseline creatinine clearance below 30 mL/min/1.73 m^2^ (calculated using the CKD-EPI equation), chronic kidney disease (CKD) stage IV or higher, end-stage renal disease, or chronic dependence on dialysis. Additional exclusion criteria included prior kidney transplantation, peritoneal dialysis at the time of AKI, and absence or incompleteness of medical record data related to renal function, such as daily and baseline serum creatinine and urea levels.

Patients were monitored from the initial exposure until the outcome (discharge, death, or transfer) for a period of up to 76 days of ICU stay, which represented the maximum length of stay observed among participants with dialysis-requiring AKI.

### 2.3. Data Collection and Surveys

For data collection, a structured questionnaire was developed and divided into three sections (patient identification, clinical variables, and ICU outcomes), based on a theoretical framework [2,6,7,20,21,22,23,24,25,26].

Part 1 consisted of patient identification items, including sex, ethnicity, date of birth, age, body mass index (BMI), type of hospital (public or private), and ICU admission date. Part 2 comprised variables such as the date of the AKI diagnosis, AKI classification (stage 1, 2, or 3), and prognostic scores including Sequential Organ Failure Assessment (SOFA), Acute Physiology and Chronic Health Evaluation II (APACHE II), and Simplified Acute Physiology Score 3 (SAPS 3), both on admission and daily. It also included the type of renal replacement therapy (RRT) modality prescribed and complications during the procedure. Additional relevant variables were also analyzed, such as risk factors for AKI development (sepsis, coronavirus disease (COVID-19), mechanical ventilation, nephrotoxic exposure), as well as underlying comorbidities, such as diabetes mellitus and arterial hypertension [6,26], surgical procedures, use of antibiotics and vasoactive drugs, and serum laboratory values including creatinine, potassium, urea, sodium, and arterial blood gases [26]. Part 3 of the questionnaire included items related to the ICU length of stay and clinical outcomes such as discharge or death, renal recovery, and dialysis dependence.

An active search was conducted to identify patients undergoing intermittent and/or continuous renal replacement therapy in the selected ICUs, followed by analysis of their electronic medical records.

Patients with AKI were classified according to the KDIGO (2012) guidelines, which stratify AKI into severity stages. Stage 1 is defined by an increase in serum creatinine of 1.5 to 1.9 times the baseline within seven days. Stage 2 is defined by a 2.0 to 2.9-fold increase in baseline serum creatinine. Stage 3 is defined by a threefold increase in serum creatinine or the initiation of dialysis [24]. Urine output was not used to assess AKI stages due to imprecision and lack of documentation on urinary volume.

Patients were classified as having dialysis-requiring AKI (AKI-D) if their electronic medical records indicated clinical conditions such as fluid overload, hyperkalemia, or metabolic acidosis requiring RRT resulting from renal or extrarenal causes. The latter was considered to be those requiring renal support therapy in situations where renal function was compromised, even if the cause of injury was not primarily renal. These patients were generally classified as stage 3 according to the KDIGO (2012) guidelines [24].

The assessment of renal function recovery was performed at different time points, i.e., at 3, 10, and 30 days, considering the KDIGO guidelines, which describe the persistence of AKI and the concept of acute kidney disease as a critical window before progression to chronic kidney disease, allowing a detailed evolutionary assessment of renal function [24,27].

### 2.4. Renal Replacement Therapy Membranes

All RRT procedures were performed using standard dialysis membranes routinely adopted in the participating intensive care units for both intermittent and continuous modalities. No adsorptive membranes, high cut-off membranes, or specific sorbent technologies designed for cytokine or inflammatory mediator removal were utilized in this cohort. Intermittent RRT was delivered with high-flux synthetic membranes (polyethersulfone or polysulfone), and continuous RRT employed standard hemofilters suitable for CRRT procedures, including filters based on polyethersulfone or similar materials. The choice of membranes was based solely on the institutional protocol for conventional renal support, without the inclusion of extracorporeal blood purification techniques aimed at immunomodulation.

### 2.5. Statistical Analysis

For statistical analysis, the data collected through the data collection instrument were entered into a Microsoft Excel spreadsheet (version 2502) using double data entry. All statistical analyses were performed using the Statistical Package for the Social Sciences (SPSS), version 26.0 (SPSS Inc., Chicago, IL, USA). Normality was assessed using the Kolmogorov–Smirnov test, and homogeneity of variances was evaluated using Levene’s test. Data were expressed as mean ± standard deviation or as median and interquartile range. Categorical variables were presented as percentages.

To compare groups regarding the clinical outcome of renal function recovery and timing of RRT initiation (early initiation < 24 h vs. delayed initiation >24 h), Student’s *t*-test or the Mann–Whitney U test was applied according to the normality of continuous variables.

Binary multiple logistic regression analysis was performed to identify predictors of clinical outcomes, including mortality and renal function recovery, based on variables with statistical significance. Model adjustments and predictive power were assessed using the likelihood ratio test, and regression coefficients were evaluated using the Wald test. The Omnibus test and the Hosmer–Lemeshow goodness-of-fit test were applied to evaluate the model’s adequacy.

Survival analyses were conducted using Kaplan–Meier curves. Furthermore, comparisons between groups, according to the timing of RRT initiation, were performed using the log-rank, Breslow, and Tarone–Ware tests. A *p*-value < 0.05 was considered statistically significant for all analyses.

## 3. Results

### 3.1. Baseline Characteristics of Patients

Out of 200 patients diagnosed with AKI, 119 progressed to the most severe stage requiring dialysis. These patients were stratified based on the timing of RRT initiation: early strategy (< 24 h after diagnosis of severe AKI; n = 39) and delayed strategy (>24 h; n = 80). The median time to RRT initiation was 0 days in the early group and 3 days in the delayed group (*p* < 0.001). Intermittent renal support therapy (IRRT) was the predominant modality in both strategies (*p* = 0.044); however, there was a tendency for IRRT to be initiated later, while continuous renal replacement therapy (CRRT) was more frequently used as an early strategy.

The baseline clinical characteristics were homogeneous between the groups, as summarized in Table 1. Among the evaluated patients, most were male (n = 70; 58.8%) and older adults (n = 85; 71.4%). Heart failure was a frequent comorbidity (n = 59; 49.6%) and also the reason for ICU admission in 21.8% of cases (n = 26). Sepsis was the main etiology of AKI (n = 60; 50.4%), followed by hypovolemia (n = 35; 29.4%).

Most patients achieved renal function recovery (n = 62; 52.1%). Early recovery (within 3 days) was significantly more frequent in the group that underwent the early RRT initiation strategy (*p* = 0.010).

### 3.2. Mortality and Renal Function Recovery

**Figure 2:**
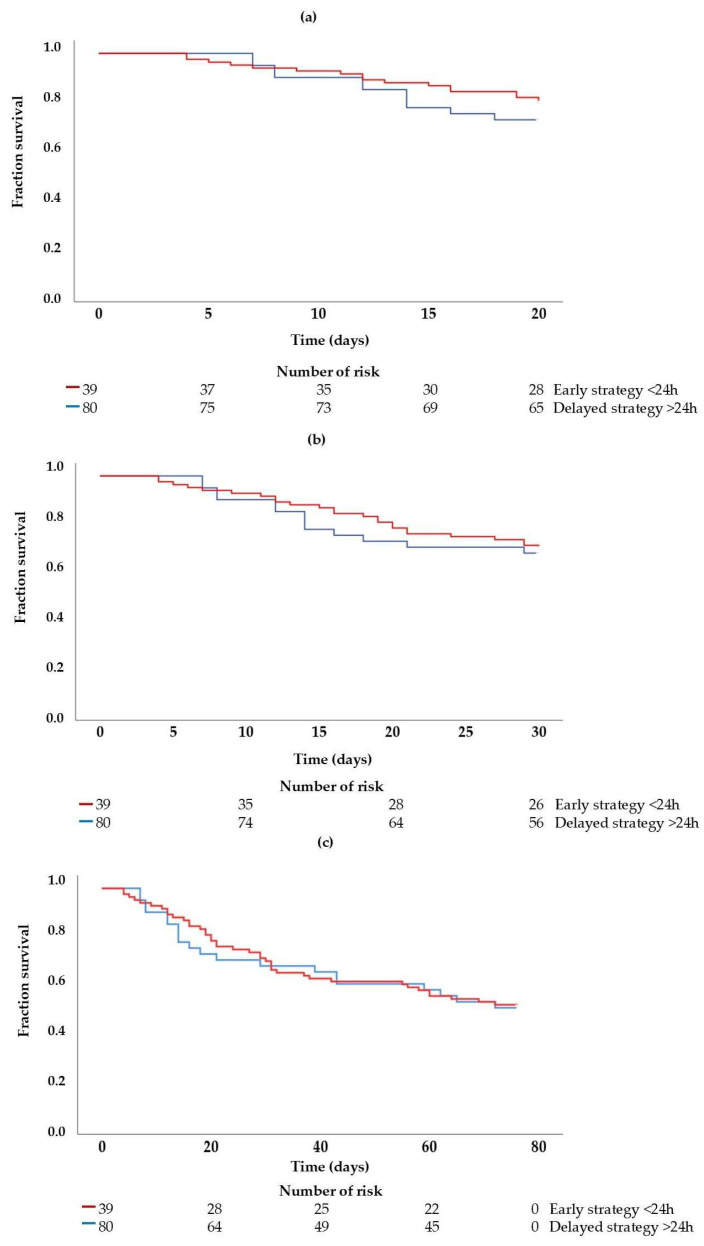
Survival curves according to the groups, early strategy < 24 h and delayed strategy >24 h, over a 20-day follow-up period (**a**), 30 days (**b**), and 76 days (**c**). Log-rank test to 20 days (**a**) *p* = 0.396; 30 days (**b**) *p* = 0.742; 76 days (**c**) *p* = 0.932. Breslow test to 20 days (**a**) *p* = 0.391; 30 days (**b**) *p* = 0.667; 76 days (**c**) *p* = 0.840. Tarone–Ware test to 20 days (**a**) *p* = 0.392; 30 days (**b**) *p* = 0.702; 76 days (**c**) *p* = 0.886.

Mortality was analyzed at three time points: 20, 30, and 76 days (total ICU length of stay). No statistically significant differences in mortality were observed between groups across these time intervals (Figure 2).

The Kaplan–Meier survival curves at 20, 30, and 76 days were closely aligned and overlapped (log-rank test: *p* = 0.396; *p* = 0.742; and *p* = 0.932, respectively). Thus, the timing of TSR initiation did not impact patient mortality, and overall survival was comparable between the early and delayed initiation groups.

The group that received early RRT and died within 30 days showed a slightly more marked initial decline; however, over time, the mortality rate between both groups converged. Therefore, hospital mortality was similar, and no clear difference in survival was observed between the groups.

A significant difference in SOFA scores was observed between strategies, with higher values in the early-initiation group (*p* = 0.016), indicating greater severity on the first ICU day. In contrast, no significant differences were found for SAPS 3 and APACHE II scores (Table 2).

Invasive mechanical ventilation was significantly more common in patients who received early-initiation RRT compared to those in the delayed group (87.2% vs. 62.5%, *p* = 0.004). A similar pattern was observed regarding the use of vasopressor agents, with a higher proportion of patients in the early group requiring vasoactive support (36 [92.3%] vs. 61 [76.3%], *p* = 0.026), indicating greater clinical severity in this group.

Despite the higher clinical severity observed in patients who underwent early-initiation RRT, there was a trend toward longer ICU stays in the delayed group compared to the early group (median of 28 vs. 17 days).

Descriptive analysis of renal recovery showed that a significantly higher proportion of patients in the early-initiation group recovered kidney function within 3 days (51.3% vs. 27.5%, *p* = 0.010). However, no significant differences in renal recovery were observed between the groups at 10 and 30 days (*p* = 0.092 and *p* = 0.450, respectively).

Early initiation was a strong predictor of renal function recovery within 3 days (Table 3). Patients who started RRT early were 3.26 times more likely to recover kidney function (OR 3.26; 95% CI: 1.37–7.75; *p* = 0.008). This association remained statistically significant in both unadjusted and adjusted analyses, indicating that early initiation of RRT is an independent factor for renal recovery in AKI.

ICU length of stay and age were also statistically significant predictors of renal recovery. Each additional day of ICU hospitalization was associated with a 3% increase in the likelihood of renal function recovery (OR 1.03; 95% CI: 1.01–1.06; *p* = 0.003). Additionally, older age was positively associated with renal recovery, with a 3% increase in the chance of recovery for each additional year of life (OR 1.03; 95% CI: 1.00–1.06; *p* = 0.038).

**Figure 3:**
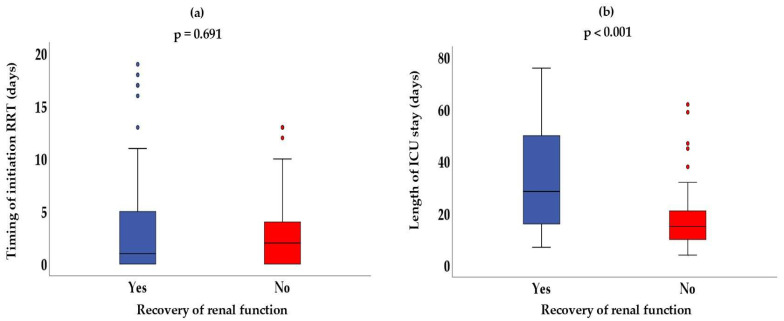
Timing of initiation of renal replacement therapy (RRT) (**a**) in days and length of intensive care unit (ICU) stay (**b**) in days, according to recovery of renal function of the patients. Colored dots represent individual outliers.

Therefore, as observed, the early initiation strategy was associated with higher rates of renal recovery. Patients who did not recover renal function generally started RRT later and had shorter ICU stays (Figure 3). The presence of outliers suggests that in some cases, RRT was initiated late, but the patients still achieved renal recovery, or conversely, early initiation did not necessarily lead to recovery.

## 4. Discussion

This prospective study evaluated whether the timing of initiating RRT influences outcomes in critically ill patients with AKI. Our findings demonstrated that the timing of RRT initiation, early (< 24 h) or delayed (>24 h), did not significantly impact mortality outcomes. Survival over an approximately 80-day follow-up period was comparable between the early and delayed RRT strategies. However, early RRT initiation appeared to offer a clinical benefit, as patients in this group showed higher rates of early renal recovery, although this was not associated with a consistent effect on mortality.

Multicenter trials such as AKIKI [11] and STAART-AKI [13] and several systematic reviews [7,14,15] did not demonstrate improved survival with early RRT initiation. In contrast, the ELAIN trial [10], a single-center randomized study involving 231 postoperative patients with lower baseline severity, reported reduced 90-day mortality with early RRT (<8 h after KDIGO stage 2). In contrast, our cohort included 119 critically ill patients with AKI who required dialysis and were therefore more clinically severe, represented at baseline by SOFA 10 (early group). Early RRT was defined as starting within 24 h after diagnosis of stage 3 in the KDIGO guidelines. The results included not only mortality (at 20, 30, and 76 days) but also renal recovery, which was an outcome not evaluated in ELAIN. These methodological and clinical differences may explain the divergent results and further reinforce the need for individualized decision-making.

The AKIKI-2 trial aimed to determine how long RRT could be safely delayed in patients with severe AKI in the absence of life-threatening complications. The more delayed strategy, applied to patients with blood urea nitrogen levels exceeding 112 mg/dL and persistent oliguria for more than 72 h, showed no clinical benefit and was associated with potential harm. Therefore, the decision to initiate or delay TSR should be individualized [12].

One of the main contributors to the high mortality observed in patients with AKI is the accumulation of uremic toxins such as urea and creatinine, along with hyperkalemia, metabolic acidosis, fluid overload, and the presence of oliguria or anuria [9,19]. In our cohort, the predominance of older adults, male, and white patients, demographic profiles often associated with increased clinical vulnerability, was accompanied by a reduced glomerular filtration rate and elevated serum urea at ICU admission, showing the need for early toxin removal. Theoretically, the prompt clearance of these toxins and excess fluid should improve clinical outcomes, including mortality and renal recovery [27]. Although prospective observational studies have shown that early RRT initiation may improve survival in patients with AKI related to trauma [28] and cardiac surgery [29,30], and one randomized trial demonstrated benefit in acute pancreatitis [31], multiple randomized controlled trials have reported consistent findings that early RRT does not reduce mortality and may even result in potential harm [14,15].

In this analysis, although early initiation of RRT had no significant impact on early or late mortality (at 20, 30, and 76 days) (log-rank: *p* = 0.396; *p* = 0.742; *p* = 0.932), it was associated with renal function recovery by the third day of therapy and emerged as an independent predictor of renal recovery.

Renal recovery, when achieved, may guide clinical decision-making in the management of critically ill patients with AKI, potentially improving prognostic outcomes [32]. Recovery has been associated with a significant reduction in hospital mortality and better long-term clinical outcomes [33]. Although in our findings renal recovery was observed early, survival did not differ between patients regardless of the initiation strategy. This underscores the importance of implementing preventive strategies, which may reduce healthcare system costs [34].

The results of our study revealed that the trend toward delayed renal function recovery (at 10 and 30 days) was more often observed in patients who initiated RRT later. Notably, these patients remained hospitalized for longer periods than those in the early strategy group (median of 28 vs. 17 days). This clinical profile appears to be more closely associated with increased mortality risk and progression to chronic kidney disease [33].

The association between delayed RRT initiation and lower renal recovery rates lies in the pathophysiological progression of AKI. Prolonged exposure to factors such as sustained hemodynamic instability, fluid overload, and an exaggerated inflammatory response contributes to irreversible structural kidney damage, including endothelial dysfunction, microvascular rarefaction, and progression to interstitial fibrosis [21]. This condition may represent a point beyond the “reversibility threshold,” where cellular injury becomes maladaptive, compromising regenerative processes. Furthermore, our data demonstrated a clear association between a higher number of organ failures, reflected by elevated SOFA scores, and impaired renal recovery. This finding reinforces the concept that multiorgan dysfunction syndrome creates a hostile biological environment, perpetuating kidney injury via systemic inflammation, oxidative stress, and reduced renal perfusion [2,35].

Nevertheless, each additional day of ICU stay was associated with a 3% increase in the likelihood of renal function recovery (OR: 1.03; 95% CI: 1.01–1.06; *p* = 0.003), possibly due to continuous intensive support and daily clinical monitoring. Although the observed lengths of stay exceeded the recommended targets established by the Brazilian Ministry of Health, ranging from 4.5 to 5.3 days according to the Hospital Quality Control Program and the Brazilian Intensive Care Medicine Association [36], prolonged ICU management may have contributed to renal recovery in patients with dialysis-requiring acute kidney injury. These findings underscore the importance of individualizing the duration of ICU stay according to clinical severity.

A retrospective study involving a large cohort of 47,903 predominantly male American adults with stage 2 and 3 AKI, defined by KDIGO criteria, demonstrated that early renal recovery (between 1 and 4 days) was associated with a lower risk of progression to kidney failure or a sustained decline ≥40% in an estimated glomerular filtration rate [37]. Similarly, in our cohort, an early RRT strategy was associated with a higher rate of renal recovery within the first few days, suggesting a more favorable prognosis for critically ill patients with dialysis-requiring AKI.

In the present study, patients who underwent early RRT exhibited greater clinical severity, including significantly higher SOFA scores (median 10 vs. 7, *p* = 0.016), more frequent use of mechanical ventilation, and a greater need for vasopressors (*p*-value = 0.042). These findings suggest that the decision to initiate early RRT may have been driven by the patients’ critical clinical condition in conjunction with rising serum creatinine levels [19]. Disease severity and its subsequent course should be taken into account to facilitate rapid and complete renal recovery [33].

Despite the clinical severity of patients in this study, no significant differences were observed between the groups in SAPS 3 and APACHE II scores (*p* = 0.952 and *p* = 0.887, respectively), suggesting that these prognostic tools may have limitations in guiding the timing of RRT initiation. This underscores the importance of individualized clinical assessment [19].

The initiation of RRT should be considered when metabolic and fluid demands exceed the total renal capacity [38]. Clinical protocols such as SCAMP (Standardized Clinical Assessment and Management Plan) have demonstrated a positive impact on reducing mortality. This tool supports the decision-making process for prescribing RRT in patients with AKI by providing objective and personalized protocols, which have been associated with improved survival outcomes [39].

In the present investigation, most patients who received early RRT underwent CRRT (*p* = 0.044), suggesting that CRRT in the context of an early strategy was more frequently associated with renal recovery. This finding was consistent with randomized trials in the field [40]. Overall, CRRT may lead to fewer hemodynamic complications and better outcomes in critically ill patients, such as improved renal function recovery [41,42,43,44,45,46], as observed in our study.

Several factors may have influenced the mortality and renal recovery outcomes observed in this study. In our cohort, the early RRT group exhibited higher clinical severity, as observed by elevated SOFA scores and a greater need for life-sustaining therapies such as vasopressors and mechanical ventilation. These indicators reflect hemodynamic instability, a known contributor to both increased mortality and impaired renal recovery in patients with dialysis-dependent AKI [47]. Additionally, the high prevalence of sepsis and cardiovascular comorbidities among our patients aligns with established risk factors for worse renal outcomes and reduced survival [48]. Persistent fluid overload, frequently observed in critically ill patients, has also been recognized as an independent predictor of higher mortality and lower renal recovery, due to its contribution to renal congestion, hypoperfusion, and progression of tubular injury [49]. Collectively, these clinical conditions may exacerbate renal ischemia and endothelial dysfunction, limiting the reversibility of organ damage and thus reducing the likelihood of renal recovery. These findings underscore the importance of individualized decision-making regarding RRT initiation, considering not only laboratory thresholds, but also the patient’s overall clinical trajectory and multi-organ involvement [11].

One of the distinctive features of our study was the detailed temporal analysis of clinical outcomes, including mortality assessed at three different time points (10, 20, and 76 days) and renal function recovery evaluated at three distinct periods, encompassing early recovery (within 3 days) and later outcomes (10 and 30 days). This approach allowed a more comprehensive understanding of the clinical progression of patients undergoing RRT.

Clinical profiles were evaluated using prognostic scores such as SOFA, SAPS 3, and APACHE II, in addition to hemodynamic, laboratory, mechanical ventilation, and vasopressor support data, enabling a thorough characterization of patient severity. The use of multivariate statistical analyses and Kaplan–Meier survival curves strengthened the consistency of the findings and enhanced their applicability in intensive care clinical practice.

This study provides meaningful contributions to existing literature by focusing exclusively on critically ill patients with dialysis-dependent AKI (KDIGO stage 3), a population characterized by high clinical complexity and elevated mortality risk. Unlike previous studies that included heterogeneous populations with varying degrees of severity, this prospective cohort study, conducted in a real-world clinical setting, specifically analyzes the impact of early initiation of RRT (< 24 h versus >24 h) on renal recovery and mortality. Moreover, it uniquely explores the interaction between the timing of initiation and different RRT modalities (intermittent, continuous, and combined), an aspect still underrepresented in the literature.

Notably, most large randomized clinical trials, such as AKIKI [11] and STARRT-AKI [13], were conducted in high-income countries. Our study contributes valuable prospective data from a lower- and middle-income country setting, where patient characteristics, ICU resources, and RRT accessibility differ substantially, thus helping to fill a critical gap in the global literature.

However, certain limitations must be acknowledged when interpreting these results. First, although the prospective design strengthens the quality of data collection, this was an observational cohort study, which limits the ability to establish causality between the timing of RRT initiation and the clinical outcomes evaluated. Therefore, the findings should be interpreted as associations rather than direct causal relationships.

The study was conducted in two specific intensive care units (ICUs) within the same geographic region, which may limit the generalizability of the results to other populations, institutions with different resources, care protocols, or epidemiological and demographic profiles. Furthermore, the relatively small sample size may have reduced the statistical power to detect subtle differences in some outcomes, such as renal recovery at 10 and 30 days.

Some of the limitations of our study that may have affected the outcomes were advanced age, decreased renal reserve and the type of membrane used. These variables may restrict the effectiveness of renal support therapy. While age is not a contraindication for renal support therapy, it is a significant factor that can impact patient outcomes, particularly in terms of survival and morbidity. The choice of dialysis membrane can also influence outcomes, although its impact may be less significant than other factors such as the patient’s overall health and the quality of care provided [50,51].

Another point that could have represented a strength in this study concerns the absence of early biomarkers of acute kidney injury, such as NGAL or KIM-1, which might have complemented the clinical assessment of AKI severity and contributed to better patient stratification. Therefore, the decision to initiate RRT may have relied on clinical, hemodynamic, and laboratory criteria, which are subject to individual interpretation by healthcare professionals and may have introduced an indication bias.

Finally, follow-up was limited to the ICU hospitalization period, which, although aligned with the study’s objective, did not allow for the assessment of long-term outcomes such as dialysis dependence or progression to chronic kidney disease. Multicenter studies with larger sample sizes, extended follow-up periods, and more controlled designs are needed to validate these findings and strengthen the evidence on the impact of early initiation of renal replacement therapy in the clinical course of critically ill patients with acute kidney injury.

## 5. Conclusions

The findings of this study indicate that the early initiation of renal replacement therapy (RRT) in critically ill patients with acute kidney injury (AKI) did not significantly affect short-term (20 and 30 days) or long-term (up to 76 days) mortality. However, early RRT was associated with higher rates of early renal recovery, particularly within the first three days after RRT initiation.

This association was statistically significant (51.3% in the early group vs. 27.5% in the delayed group; *p* = 0.010) and identified as an independent predictor in multivariate analysis (OR 3.26; 95% CI:1.37–7.75; *p* = 0.008). This finding is clinically relevant, especially considering that patients in the early group exhibited greater clinical severity at ICU admission, suggesting that timely initiation of RRT may promote renal recovery even in unfavorable clinical conditions without increasing the risk of death. Therefore, the decision to initiate RRT should be guided by objective clinical criteria, such as organ dysfunction scores, the need for ventilatory support, and the use of vasopressors, rather than by isolated laboratory markers.

These results highlight the importance of an individualized and evidence-based approach to severe AKI management and reinforce the need for multicenter, randomized trials with larger sample sizes to validate and generalize the observed outcomes.

## Figures and Tables

**Table 1:** Baseline characteristics of patients (n = 119).

Variables	All Patients (n = 119)	Early Strategy < 24 h (n = 39)	Delayed Strategy >24 h (n = 80)	*p*-Value
Demographics characteristics				
Age (years)	71 [54–80]	72 [59–80]	68 [53–80]	0.766
Male, n (%)	70 (58.8)	23 (59.0)	47 (58.8)	0.571
Patients + 60, n (%)	85 (71.4)	28 (71.8)	57 (71.3)	0.585
BMI (kg/m^2^)	27.6 ± 6.9	27.5 ± 6.2	27.8 ± 7.4	0.414
Current smokers, n (%)	17 (14.3)	8 (20.5)	9 (11.3)	0.141
Ethnicity, n (%)				
White	64 (53.8)	17 (43.6)	47 (58.8)	
Black	8 (6.7)	4 (10.3)	4 (5.0)	0.231
Others	47 (39.5)	18 (46.2)	29 (36.3)	
Comorbidities, n (%)				
Diabetes	44 (37.0)	11 (28.1)	33 (41.3)	0.118
Hypertension	56 (47.1)	20 (51.3)	36 (45.0)	0.327
Heart failure	59 (49.6)	22 (56.4)	37 (46.3)	0.199
COPD	12 (10.1)	4 (10.3)	8 (10.0)	0.599
Cancer	19 (16.0)	3 (7.7)	16 (20.0)	0.069
COVID-19	8 (6.7)	5 (12.8)	3 (3.8)	0.075
CKD 1–3	48 (40.3)	14 (35.9)	34 (42.5)	0.313
Admission category, n (%)				
Medical	86 (72.3)	26 (66.7)	60 (75.0)	0.230
Surgery	33 (27.7)	13 (33.3)	20 (25.0)	
Categories of ICU admission, n (%)				
Sepsis	25 (21.0)	10 (25.6)	15 (18.7)	
Surgery	8 (6.7)	0	8 (10.0)	
Cardiovascular	26 (21.8)	8 (20.5)	18 (22.5)	
GI	11 (9.2)	5 (12.8)	6 (7.5)	
Pneumology	14 (11.8)	5 (12.8)	9 (11.3)	
Neurological	6 (5.0)	0	6 (7.5)	
Oncology	12 (10.1)	2 (5.1)	10 (12.5)	
Others	17 (14.4)	9 (23.2)	8 (10.0)	
Cause of AKI, n (%)				
Sepsis	60 (50.4)	23 (59.0)	37 (46.3)	
Hypovolemia	35 (29.4)	11 (28.1)	24 (30.0)	
Obstructive nephropathy	6 (5.0)	1 (2.6)	5 (6.2)	
Nephrotoxicity	16 (13.4)	3 (7.7)	13 (16.3)	
Glomerulonephritis including NS	2 (1.8)	1 (2.6)	1 (1.2)	
Clinical data				
Serum urea (mmol/L)	79.6 [44.8–125.5]	78.0 [39.6–147.0]	79.6 [48.4–125.0]	0.855
Serum Cr basal (mg/dL)	1.1 [0.9–1.4]	1.2 [0.9–1.3]	1.1 [0.9–1.4]	0.668
Clearance of Cr (mL/min)				
Basal	70.0 ± 24.9	67.7 ± 23.8	71.5 ± 25.8	0.878
Admission	29 [20–58]	30 [19–52]	29 [20–61]	0.861
Discharge/death in the ICU	33 [22–74]	33 [22–69]	35 [20–87]	0.388
Serum potassium (mmol/L)	4.3 ± 1.0	4.4 ± 1.0	4.3 ± 1.0	0.933
Serum sodium (mmol/L)	138 [134–141]	139 [135–143]	137 [134–141]	0.095
Hematocrit (mL/dL)	33.0 ± 8.5	33.2 ± 8.0	32.9 ± 8.8	0.444
Renal replacement therapy				
IRRT, n (%)	61 (51.3)	16 (41.0)	45 (56.2)	
CRRT, n (%)	15 (12.6)	9 (23.1)	6 (7.5)	0.044
IRRT & CRRT, n (%)	43 (36.1)	14 (35.9)	29 (36.3)	
Timing of initiation (days)	1 [0–5]	0 [0]	3 [2–8]	<0.001
Outcomes in the ICU, n (%)				
All-cause mortality, n (%)				
20 days	28 (23.5)	11 (28.2)	17 (21.3)	0.268
30 days	38 (31.9)	13 (33.3)	25 (31.3)	0.489
76 days	61 (51.3)	20 (51.3)	41 (51.2)	0.576
Recovery of renal function, n (%)	62 (52.1)	23 (59.0)	39 (48.8)	0.197
At 3 days	42 (35.3)	20 (51.3)	22 (27.5)	0.010
At 10 days	18 (15.1)	3 (7.7)	15 (18.9)	0.092
At 30 days	2 (1.7)	0	2 (2.5)	0.450

BMI: body mass index; COPD: choronic obstructive pulmonary disease; CKD: chronic kidney disease; ICU: intensive care unit; GI: gastrointestinal; AKI: acute kidney injury; NS: nephritic syndrome; Cr: creatinine; IRRT: intermittent renal replacement therapy; CRRT: continuos renal replacement therapy; IRRT & CRRT: intermittent renal replacement therapy and continuos renal replacement therapy. Categorical data are expressed as percentage and continuous data as median (interquartile ranges). Chi square test or Fisher test, Student’s *t*-test and Mann–Whitney test.

**Table 2:** Severity scores and clinical support measures at ICU admission in patients with dialysis-requiring AKI (n = 119).

Variables	All Patients (n = 119)	Early Strategy < 24 h (n = 39)	Delayed Strategy >24 h (n = 80)	*p*-Value
Length of ICU stay (days)	24 [14–44]	17 [13–40]	28 [16 –50]	0.838
SAPS 3 at ICU admission (score)	60.61 ± 18.75	60.0 ± 20.5	61.0 ± 17.8	0.952
SAPS 3 mortality prediction (%)	39.8 [9.9–61.3]	28.7 [9.2–64.9]	39.8 [13.3–60.4]	0.919
SOFA at first day (score)	7 [3–12]	10 [3–13]	7 [3–11]	0.016
SOFA mortality prediction, n (%)				
<10%	69 (58.0)	16 (41.0)	53 (66.2)	
10–20%	15 (12.6)	5 (12.8)	10 (12.5)	
20–50%	15 (12.6)	7 (18.0)	8 (10.0)	
50–70%	14 (11.8)	8 (20.5)	6 (7.5)	
>70%	6 (5.0)	3 (7.7)	3 (3.8)	
APACHE II at first day (score)	14 [11–18]	13 [10–19]	14 [11–19]	0.887
APACHE II mortality prediction (%)	15.0 [14.8–29.2]	15.0 [14.9–29.6]	18.6 [14.6–32.2]	0.755
Respiratory support (yes), n (%)	84 (70.6)	34 (87.2)	50 (62.5)	0.004
Duration of respiratory support (days)	12 [7–24]	10 [7–18]	12 [67–26]	0.399
Vasoactive drugs (yes), n (%)	97 (81.5)	36 (92.3)	61 (76.3)	0.026
Vasoconstriction, n (%)	92 (77.3)	33 (84.6)	59 (73.8)	0.042

ICU: intensive care unit; SAPS: Simplified Acute Physiology Score; SOFA: Sequential Organ Failure Assessment; APACHE II: Acute Physiology and Chronic Health Evaluation II. Categorical data are expressed as percentages and continuous data as median (interquartile ranges). Chi square test or Fisher test, Student’s *t*-test and Mann–Whitney test.

**Table 3:** Risk factors to recovery of renal function of the critically ill patients admitted to the intensive care units (n = 119).

Risk Factors		Unadjusted			Multivariate	
	Β	Odds Ratio (95% CI)	*p*-Value	Β	Odds Ratio (95% CI)	*p*-Value
Early strategy < 24 h (yes)	1.24	3.45 (1.37–8.69)	0.008	1.81	3.26 (1.37–7.75)	0.008
Length of ICU stay (days)	0.04	1.04 (1.01–1.06)	0.004	0.04	1.03 (1.01–1.06)	0.003
Age (years)	0.03	1.03 (1.00–1.06)	0.046	0.03	1.03 (1.00–1.06)	0.038
Respiratory support (yes)	0.53	0.59 (0.15–2.34)	0.454			
Vasoactive drugs (yes)	0.36	1.43 (0.32–6.47)	0.641			
SOFA on first day (score)	0.01	1.01 (0.91–1.12)	0.854			

CI: confidence interval; ICU: intensive care unit; SOFA: Sequential Organ Failure Assessment.

## Data Availability

Data are contained within the article. Additional data may be available upon reasonable request from the corresponding author.

## Abbreviations

The following abbreviations are used in this manuscript:

AKI	acute kidney injury
AKI-D	dialysis-requiring acute kidney injury
CKD	chronic kidney disease
ESKD	end-stage kidney disease
ICU	intensive care unit
IRRT	intermittent renal replacement therapy
CRRT	continuous renal replacement therapy
KDIGO	Kidney Disease: Improving Global Outcomes
RRT	renal replacement therapy
SAPS 3	Simplified Acute Physiology Score 3
SOFA	Sequential Organ Failure Assessment
APACHE II	Acute Physiology and Chronic Health Evaluation II
ROC	receiver operating characteristic
SPSS	Statistical Package for the Social Sciences
STROBE	Strengthening the Reporting of Observational Studies in Epidemiology
COVID-19	Coronavirus disease 2019
